# Medico-Legal

**Published:** 1896-12-19

**Authors:** 


### Medico-Legal.

### BREAY v. BROWNE.

In the Queen's Bench Division, on December 15th, the appeal brought by Sir James Crichton Browne against a decision of the Judge of the City of London Court in the action recently brought against him by Miss Breay, a member of the Royal British Nurses' Association, was concluded. It will be remembered that at the trial the plaintiff was awarded one farthing damages, and judgment was given in her favour, with costs on the higher scale. On Tuesday it was ruled by Mr. Justices Wills and Wright that there was no precedent for the action, and no evidence of malice. The appeal was therefore allowed, with costs, and leave to further appeal refused.

### STIVEN v. WELSFORD.

The action for libel brought by Dr. Stiven, of Harrow, against Dr. Welsford, of Dover, in regard to certain statements made by the latter charging Dr. Stiven with negligence and ignorance in the treatment of Dr. Welsford's brother, one of the masters at Harrow, was concluded on December 16th, when the jury found for the plaintiff—damages £75.

